# Genetic Characterization of *Myf5* and *POU1F1* Genes in Different Egyptian Local Rabbit Breeds and Their Association with Growth Traits

**DOI:** 10.1007/s10528-023-10604-5

**Published:** 2023-12-21

**Authors:** Sahar Ahmed, Neama Ibrahim Ali, Hassan Ramadan Darwish, Lamiaa Mohamed Salem, Reda Ismail Elsayad, Amira El-Keredy

**Affiliations:** 1 Department of Cell Biology, National Research Centre, Biotechnology Research Institute, Giza, Egypt; 2 Department of Genetics, Faculty of Agriculture, Tanta University, Tanta, Egypt

**Keywords:** Native rabbit breeds, Growth traits, Genetic diversity, Myogenic regulatory factor 5 (*MYf5*) gene, POU class 1 homeobox 1 (*POU1F1*)

## Abstract

Genetic characterization and its association with quantitative traits in local breeds are important tools for the genetic improvement and sustainable management of animal genetic resources. Myogenic regulatory factor 5 (*MYf5*) and POU class 1 homeobox 1 (*POU1F1*) are candidate genes which play important roles in growth and development of mammals. The present study aims to detect the genetic diversity of the *MYf5* and *POU1F1* genes in four local Egyptian rabbit breeds and their association with growth traits, using PCR-restriction enzyme (PCR–RFLP), PCR-single-strand conformational polymorphism (PCR–SSCP), and direct sequencing techniques. The results showed that *MYF5* exon 1 was observed with two genotypes in Baladi Black (BB), Gabali (GB) and New Zealand White (NZW) breeds while APRI-line (APRI) presented one genotype. The genetic diversity of *Myf5* exon 2 between breeds showed two genotypes in APRI compared to three in NZW and four genotypes in BB and GB breeds. The genetic diversity of the *POU1F1* gene (intron 5 and partial cds) in different rabbit breeds was two genotypes in NZW and three genotypes in BB, GB, and APRI breeds with different frequencies for each genotype. Based on the statistically significant difference between genes genotypes and growth weight, the results suggested that the genotypes of *Myf5* exon 2 (1 and 2) of the BB breed, *Myf5* exon 2 genotype 2 of the APRI breed, and genotype 1 of *Myf5* exon 1 and genotype 1 of *POU1F1* of the NZW breed compared to genotypes for each gene can be considered candidate molecular markers associated with the improvement of growth traits in these breeds.

## Introduction

Rabbit growth traits are still improving based on the heritability and crossbreed between local and exotic breeds which is reflected in the genetic flow of local breeds that are acquired through adaptation to the surrounding environment and diseases resistance. Studying the genotypes of different traits of local breeds is considered an important key toward improving animals genetic and conservation of the genetic resources and sustainable management of adapted local breeds (Abdel-Kafy et al. 2018; Ramadan et al. 2020). Therefore, the marker-assisted selections (MAS) process has become an important tool for animal genetic improvement and sustainable management of genetic resources using advanced molecular tools to select the traits of interest based on a marker (morphological, biochemical or DNA/RNA variation) linked to the trait of interest (e.g., productivity, reproduction, and disease resistance) (FAO 2007, 2011; Boettcher et al. 2022).

Quantitative traits are polygenic which means that they are controlled by many genes and gene interactions. Candidate genes analysis is the tool of the quantitative traits for genetic improvements; it is reported as a successful application in identifying several DNA markers associated with production traits (Khalil 2020).

Myogenic regulatory factor 5 (*MYf5*) gene plays an important role in muscle growth and development. The gene is involved in the skeletal muscle cell development during embryogenesis (Rawls et al. 1995; Braun and Arnold 1996; Sabourin and Rudnicki 2000; Seong et al. 2011; Zhang et al. 2022). In different mammalian, the *MYf5* proteins are reported to be highly conserved (Zhao et al. 2020(. In cattle, the *MYF5* gene polymorphism has been described to be associated with growth traits (Seong et al. 2011; Li et al. 2004; Chung and Kim 2005; Zhang et al. 2007). Nasr et al. (2016) recorded three genotypes of the *MYf5* gene (AA, AB, BB) in the Friesian bull calves; with a higher body weight for the AB genotype than that for BB and AA genotypes. The SNPs analysis of *MYf5* of Qinchuan cattle revealed three novel SNPs at 5785C > T, g.5816A > G in the 3rd exon region, and g.6535A > G in the 3’ UTR were associated with growth performance and beef quality traits (Zhao et al. 2020).

POU class 1 homeobox 1 (*POU1F1*) is one of the candidate genes acting as a regulator for growth hormone (*GH*), prolactin (PRL) and thyroid-stimulating hormone β (*TSHβ*) by binding to target DNA promoters as a dimer in mammalian animals (Lan et al. 2007a,b, 2009; Daga et al. 2013; Feng et al. 2012; Bai et al. 2016). The genetic polymorphisms of the *POU1F1* gene have been associated in cattle with milk yield, milk composition, milk production traits (Huang et al. 2008; Zakizadeh et al. 2007; Ahmadi et al. 2015), and carcass weight (Pan et al. 2008; Seong et al. 2011; Wang et al. 2015). In sheep and goats, it was reported as a candidate gene associated with growth, weaning weight, litter size, milk production, and meat quality traits (Mura et al. 2012; Ozmen et al. 2014; Jalil-Sarghale et al. 2014; Sadeghi et al. 2014; Bai et al. 2016; AL-Khuzai and AL-Anbari 2018, Jaffar et al.^2019^). In goats, cattle, and sheep, the POU1F1 gene is 6 exons and 5 introns located on the 1q21–22 chromosome (Woollard et al. 2000) while it is mapped to chromosome 14 in rabbits, consisting of seven exons and six introns (Wang et al. 2015).

The current study aims to detect the genetic diversity of the *MYf5* and *POU1F1* genes in four local Egyptian rabbit breeds (Baladi Black, APRI-line, Gabali) and adapted New Zealand White breed and to investigate their possible association with growth traits, using PCR-restriction enzyme (PCR–RFLP), PCR-single-strand conformational polymorphism (PCR–SSCP), and direct sequencing techniques.

## Materials and Methods

### Rabbit Breeds

The present study was carried out on Baladi Black, APRI-line, New Zealand White, and Gabali rabbit breeds. The Baladi Black (BB) breed has been derived from crossing native breed with an exotic Flemish Giant breed since 1950. The APRI-line was founded by mating Baladi Red (BR) bucks to V-line does, obtaining the F1, F2, and then F3, starting the selection at this generation (Youssef et al. 2008). APRI rabbits are Egyptian line selected for litter weight at weaning according to Abou Khadiga et al. (2010). The New Zealand White breeds were brought to Egypt from Poland in 1964. Gabali breed is found in the north Mediterranean coast (western desert) and Sinai Peninsula.

### Genetic Analysis

#### Samples

Eighty blood samples were collected for each breed from ear vein. The genomic DNA extraction was performed according to John et al. 1991. The samples represented rabbit at age 12–15 weeks from each breed. The body weight was taken for each animal during the blood sample taken using a digital balance.

### Primer Design and PCR Amplification

The primers for the gene *Myf5* exon 1 (1287-bp) and *Myf5* intron 1 and exon 2 (940-bp) were designed using Primer 3.0 software (Applied Biosystems, USA) while the primers used for *POU1F1* were according to Wang et al. (2015) (Table 1). The thermal cycling program was 5 min at 95 °C, followed by 35 cycles of denaturation at 95 °C for 45 s, annealing temp. according to each gene in Table 1, extension at 72 °C for 1 min, and final extension at 72 °C for 7 min. The PCR products were loaded on 1.5% agarose gels (with 100-bp DNA ladder) for electrophoresis separation to gauge the success of PCRs. The gels were visualized under UV and photographed using Gel documentation system (Bio-Rad Laboratories, Hercules, CA, USA).

**Table 1:** Primer sequences and characteristics of amplified PCR products

Name	Sequence	PCR Fragment size	Annealing Temp ( °C)	RE
*Myf5A*	F: CAGGCAACTGCCCTTGTTAATT R: TGCTTGGTTTAGGAAGGCTCAG	1287-bp (exon 1)	67	*Hind III*
*Myf5B*	F: CTGCTGCTCTCCTCTTCATCAAG R: GTAACAGGCCTGGAAGAACTGATC	940-bp (exon 2)	62.5	*Hae III*
*POU1FI*	F: GCTGGAGGAAGCTGAGCAAGT R: GAATACCTTATGGTCGTCCTCCG	798-bp (intron 5 and partial cds)	67	*EcoR I*

### PCR–RFLP Analysis

Eight μl of PCR product for each fragment was digested with 10 units of specific restriction enzyme (Table 1) in a final reaction volume 20 μl. The reaction mixture was incubated at 37 °C in water bath for 4 h. After restriction digestion, the restricted fragments were analyzed by electrophoresis on 2.5% agarose/1X TBE gel stained with ethidium bromide. The 100-bp ladder was used as molecular size marker. The bands were visualized by gel documentation ((Bio-Rad Laboratories, Hercules, CA, USA). The restriction enzymes were used based on the sequences of *Myf5* exon 1 (1287-bp), *Myf5* intron 1 and exon 2 (940-bp), and *POU1F1* (798-bp) from GenBank database and Restriction Mapper software version 3.

### Single-Strand Confirmation Polymorphism (SSCP) Analysis

A quantity of 5 µl of each amplicon was mixed with 15 µl of denaturing solution (98% formamide, 10 mM NaOH, 0.05% bromophenol blue, and 0.05% xylene cyanol); then the mixture was denatured by heating at 96 °C for 7 min and immediately chilled on ice for 10 min (Gasser et al. 2006). The denaturized samples were loaded on 8% polyacrylamide gel (acrylamide: bisacrylamide 37.5: 1 v/v) using 20 cm × 20 cm vertical unit in 1 × TBE buffer. Electrophoresis was carried out at a constant voltage of 280 V at 4 °C for 16 h. Then after, gels were stained in ethidium bromide solution at 0.5 mg/ml for 5 min, washed with distilled water, and visualized under UV light and photographed using gel documentation system (Bio-Rad Laboratories, Hercules, CA, USA).

### Direct Sequencing Analysis

The PCR products of the amplified fragments were cut from the agarose gel and purified by purification kit (Invitrogen kit, USA), following the manufacturer’s instruction. The purified PCR products were sent for sequencing to Macrogen Incorporation (Seoul, South Korea), and primers for sequencing were similar to those used for the PCR-amplification.

### Bioinformatics and Statistical Analysis

The obtained sequences were confirmed using BLAST software (https://blast.ncbi.nlm.nih.gov/ Blast.cgi). The sequence’s results were submitted to GenBank. Sequences were aligned, inspected and trimmed using GENEIOUS 6.0 software (New Zealand; Kearse et al. 2012), and exported in FASTA format for haplotype diversity (h) and nucleotide diversity (π) were determined, from DnaSP6 software (Rozas et al. 2003). Statistically significant difference was determined using one-way ANOVA with post hoc Tukey HSD (honestly significant difference) procedure that facilitates pairwise comparisons. F statistic and the p-value (p ≤ 0.05) were calculated.

## Results

### Genetic Variation of *Myf5* Gene *(*exon 1)

**Fig. 1:**
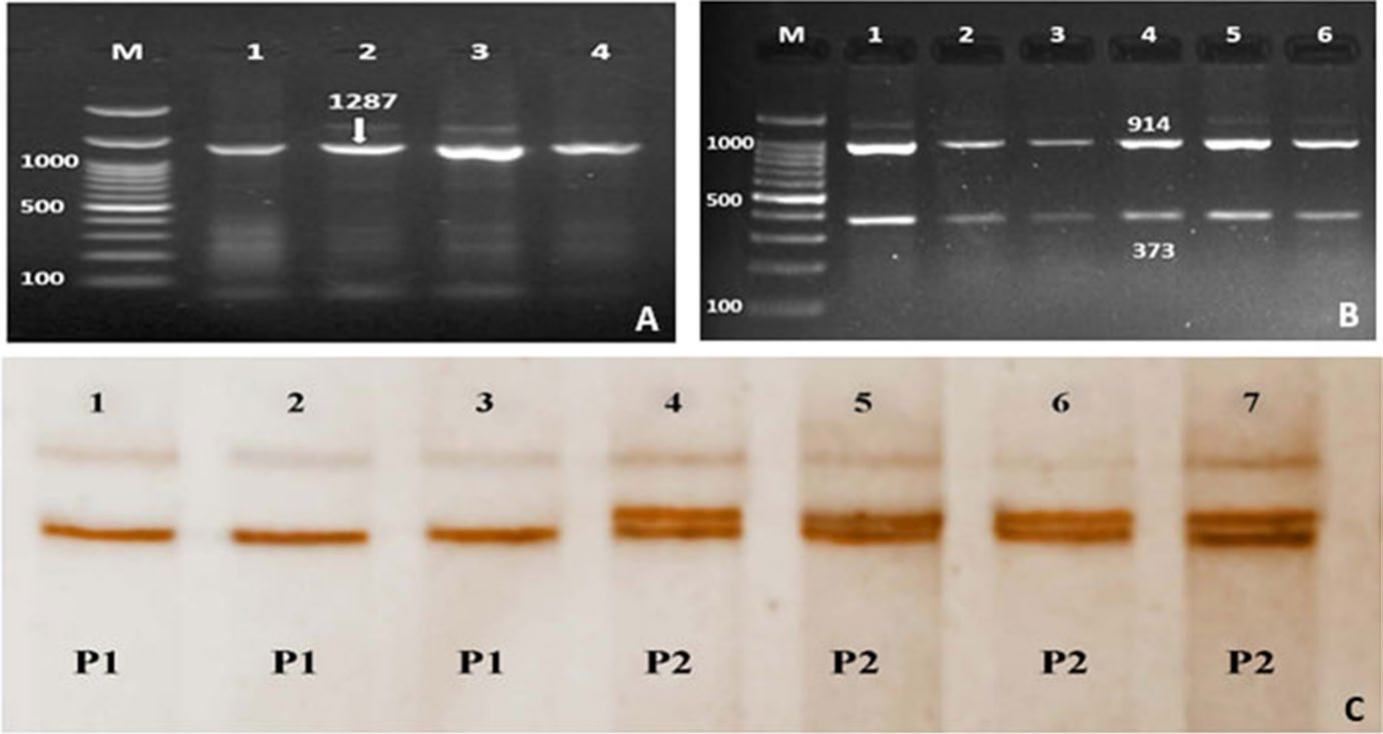
**A** PCR-amplified products representing *Myf5* gene *(*exon 1). Lane M: 100-bp DNA marker; Lanes (1–4) represent the PCR products band size at 1287-bp. **B** PCR–RFLP fragments from lane 1–8, Lane M: 100-bp DNA marker; **C** PCR-SSCP analysis, PCR-SSCP analysis, P1: Lanes 1–3, P2 Lane 4–7

The successful PCR fragments of *Myf5* gene (exon 1) amplified 1287-bp are shown in Fig. 1A. All the PCR products digested by restriction enzyme *HindIII* produced two restriction fragments at 914-bp and 373-bp (Fig. 1B). The SSCP analysis revealed two variant patterns for the PCR products (P1 and P2, Fig. 1C). The SSCP patterns reflect the *Myf5* gene (exon 1) genotypes as P1 genotype 1 and P2 genotype 2.

**Fig. 2:**
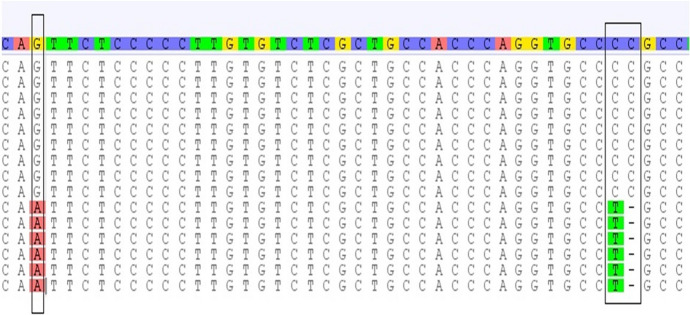
The SNPs substitution of *Myf5* exon 1

The sequence alignment of *Myf5* exon 1 was documented in GenBank accession numbers OK663024 and OK663025. The SNPs substitutions of Myf5 exon 1 represent A > G, T > G,—> C, A > G of genotype 1 and G > A, G > T, C > -, G > A of genotype 2 as shown in Fig. 2. The results from the DnaSP6 software analysis were 0.00475 for the variance of haplotype diversity and 0.00256 ± : 0.00034 for nucleotide diversity (π).

Table 2 shows the frequency of the *Myf5* exon 1 genotypes in different rabbit breeds. The results revealed that the genotype 1 was observed with high frequency in BB, APRI, and NZW breeds. APRI breed showed only genotype 1. GB breed showed a high frequency of 60% for genotype 2 compared to 40% for genotype 1. The genotypes of *Myf5* exon 1 association with body weight indicated that there were no significant differences between genotypes 1 and 2 in BB, GB, and APRI breeds. The NZW breed displayed significant differences between genotypes 1 and 2. The mean value of body weight for genotypes 1 and 2 between breeds ranged from 3.00 ± 0.20 kg to 3.19 ± 0.55 kg and 2.59 ± 0.24 kg to 2.9 ± 0.24 kg, respectively, without significant differences.

**Table 2:** Frequency of the *Myf5* gene genotypes (exon 1) in different rabbit breeds and their associated body weight

Breed	Genotyp1		Genotyp2	
	Weight/kg Mean ± SD	Frequency %	Weight/kg Mean ± SD	Frequency %
BB	3.17 ± 0.57	58.7	2.9 ± 0.24	41.3
GB	3.19 ± 0.55	40	2.84 ± 0.11	60
APRI	3.11 ± 0.33	100	–	–
NZW	3.00 ± 0.20^A^	60	2.59 ± 0.24^B^	40

The rabbit breeds: Baladi Black (BB), Gabali (GB), APRI-line (APRI), and New Zealand (NZW)

(A and B) in the same row significantly different at p ≤ 0.05

### Genetic Variation of* Myf5 *Gene* (*exon 2)

**Fig. 3:**
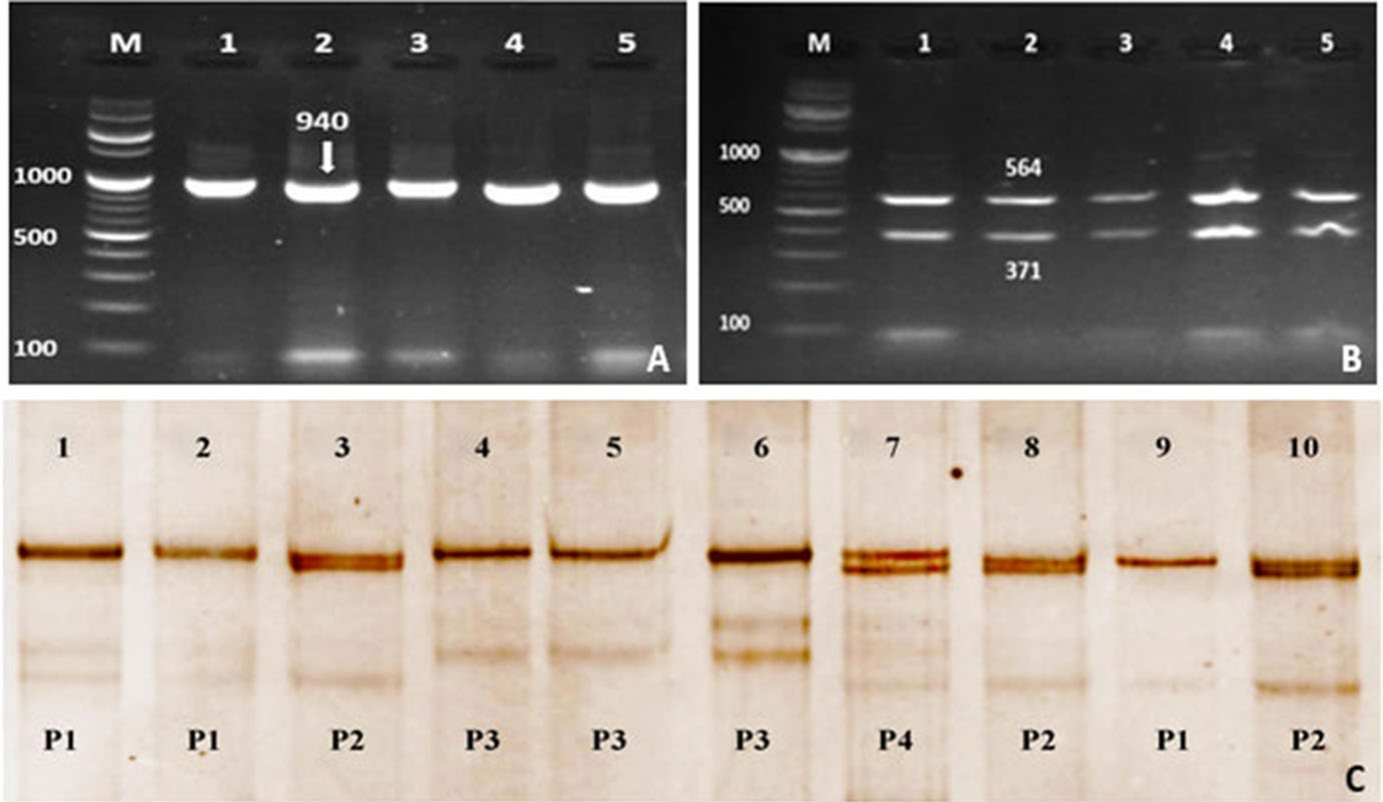
**A** PCR-amplified products representing *Myf5* gene* (*exon 2). Lane M: 100-bp DNA marker; Lanes (1–5) represent the PCR products band size at 940-bp. **B** PCR–RFLP fragments from lane 1 to 5, Lane M: 100-bp DNA marker; **C** PCR-SSCP analysis, P1: Lanes 1, 2, and 9, P2 Lane 3, 8 and 10, P3 Lanes 4, 5, and 6, P4 Lane 7

The successful PCR products of *Myf5* gene* (*exon 2) amplified 798-bp are presented in Fig. 3A. The PCR products digested by restriction enzyme (*HaeIII*) showed two fragments at 564-bp and 371-bp for all the PCR products (Fig. 3B). The SSCP analysis detected four variant patterns for the PCR products (P1, P2, P3, and P4, Fig. 3C). The SSCP patterns reflect the *Myf5* gene* (*exon 2) genotypes as P1 genotype 1, P2 genotype 2, P3 genotype 3, and P4 genotype 4.

**Fig. 4:**
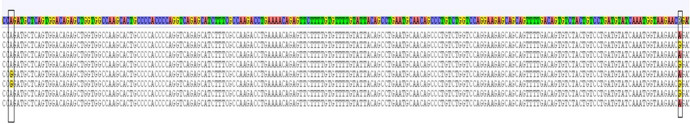
The SNPs substitution of *Myf5* exon 2

The sequence alignment for *Myf5* exon 2 was documented in GenBank accession numbers OK663026, OK663027, OK663028, and OK663029. The SNPs substitutions of *Myf5* exon 2 represent A/A for genotype1, A/G for genotype 2, G/A for genotype 3, and G/G for genotype 4 (Fig.4). The results from the DnaSP6 software were 0.03125 for the variance of haplotype diversity and 0.00366 ± 0.00079 for nucleotide diversity (π).

The frequency of the *Myf5* exon 2 genotypes in different rabbit breeds shows that the BB and GB breeds were with four genotypes with high frequency for genotype 1 compared to the other three genotypes (Table 3). APRI breed showed two genotypes (2 & 4) with a high frequency for genotype 2 (85%) compared to genotype 4 (15%) while the NZW breed recorded three genotypes (1, 2 & 4) with a high frequency for genotype 1 compared to other genotypes. Genotype 4 was the lowest frequency between breeds.

**Table 3:** Frequency of the *Myf5* gene genotypes (exon 2) in different rabbit breeds and their associated body weight

Breed	Genotype1		Genotype2		Genotype3		Genotyp4	
	Weight/kg Mean ± SD	Frequency %	Weight/kg Mean ± SD	Frequency %	Weight/kg Mean ± SD	Frequency %	Weight/kg Mean ± SD	Frequency %
BB	2.82 ± 0.44^Aa^	51.3	2.85 ± 0.49^A.a^	28.7	2.27 ± 0.22^Ba^	13.7	2.27 ± 0.20^Ba^	6.3
GB	3.12 ± 0.26^Aa^	50	3.35 ± 0.26^A.a^	20	3.5 ± 0.36^Ab^	23.8	3.2 ± 0.3^Ab^	6.2
APRI	–	–	3.13 ± 0.41^A.a^	85	–	–	2.73 ± 0.14^Bab^	15
NZW	2.73 ± 0.29^Aa^	41.3	2.69 ± 0.44^A.a^	25	–	–	3.10 ± 0.39^Ab^	33.7

The rabbit breeds: Baladi Black (BB), Gabali (GB), APRI-line (APRI), and New Zealand (NZW)

The small letters (a and b) in the same column significantly different at p ≤ 0.05. The capital letters (A and B) in the same row significantly different at p ≤ 0.05

The association of *Myf5* exon 2 genotypes with body weight revealed significant differences between genotypes (1 and 2) and genotypes (3 and 4) in BB breed. In APRI breed, genotypes 2 and 4 showed significant differences while GB and NZW breeds showed no significant differences between genotypes. The mean value of body weight for genotypes 1 and 2 between breeds ranged from 2.73 ± 0.29 kg to 3.12 ± 0.26 kg and 2.69 ± 0.44 kg to 3.35 ± 0.26 kg, respectively, without statistically significant differences. The mean value of body weight for genotype 3 showed significant differences between breeds BB (2.27 ± 0.22 kg) and GB (3.5 ± 0.36 kg). The mean value of body weight for genotype 4 between BB (2.27 ± 0.20 kg) and GB (3.2 ± 0.3 kg) breeds and also between BB (2.27 ± 0.20 kg) and NZW (3.10 ± 0.39 kg) breeds showed significant differences.

### Genetic Variation of *POU1F1* Gene (Intron 1 and Exon 1)

**Fig. 5:**
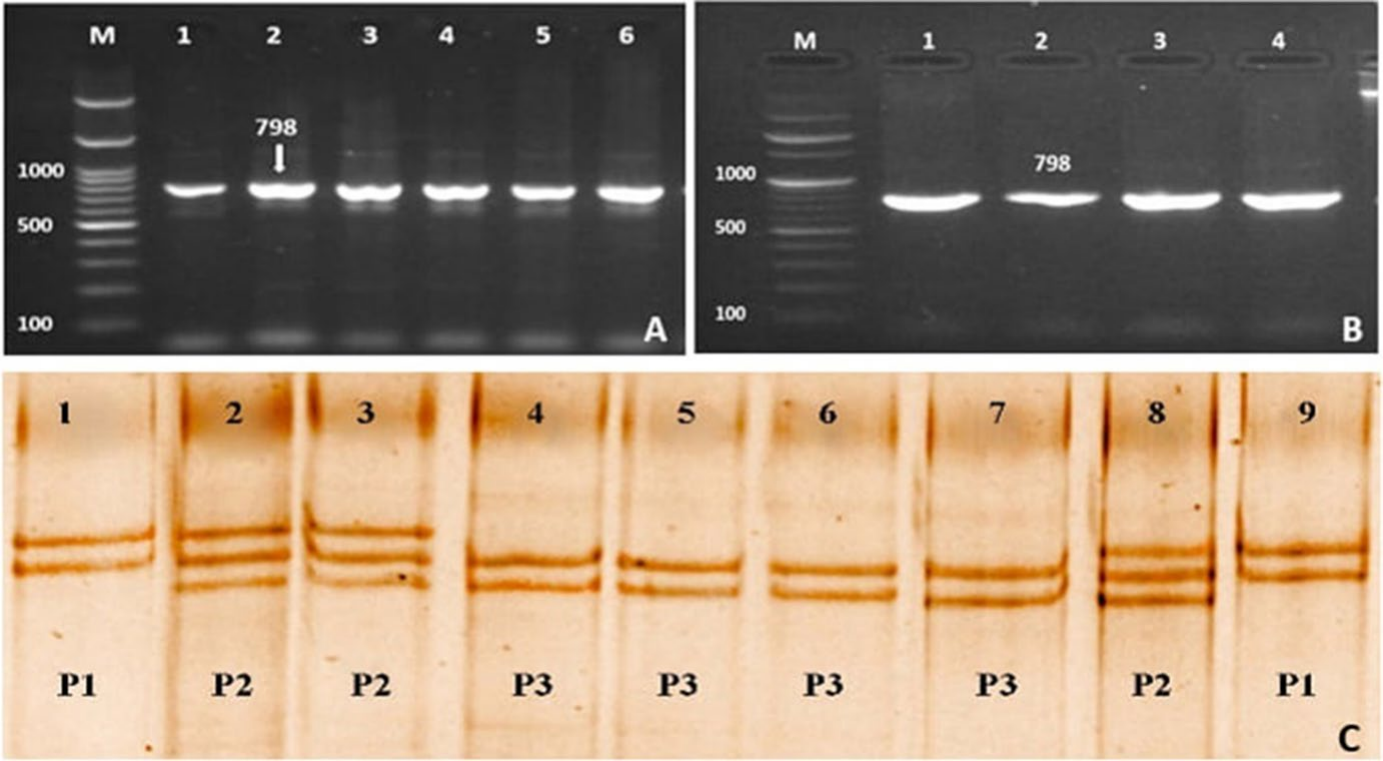
**A** PCR amplified products representing of *POU1F1* gene (intron 5). Lane M: 100-bp DNA marker; Lanes (1–5) represent the PCR products band size at 780-bp. **B** PCR–RFLP fragments from lane 1 to 4, Lane M: 100-bp DNA marker; **C** PCR-SSCP analysis, P1: Lanes 1 and 9, P2 Lane 2, 3, and 8, P3 Lanes 4–7

The successful PCR products of *POU1F1* gene (intron 5 and partial cds) amplified 987-bp are shown in Fig. 5A. The restriction enzyme (*EcoRI*) showed an uncut fragment in all the PCR products (Fig. 5B). The SSCP analysis showed three variant patterns for the PCR products (P1, P2 and P3, Fig. 5C). The SSCP patterns reflect the *POU1F1* gene genotypes as P1 genotype 1, P2 genotype 2. and P3 genotype 3.

**Fig. 6:**
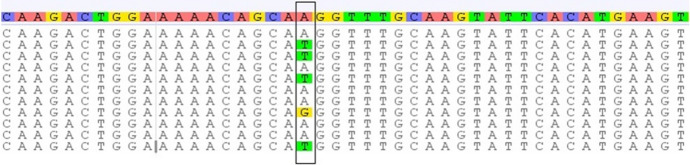
The SNPs substitution of *POU1F1* intron 1 and exon 1

The sequence alignment for *POU1F1* products was documented in GenBank accession numbers OK663030, OK663031, and OK663032. The SNPs substitution of *POU1F1* intron 5 and partial cds represents A > G > T for genotype 1, T > G > A for genotype 2, and G > T > A for genotype 3 (Fig. 6). The results from the DnaSP6 software were 0.01078 for the variance of haplotype diversity and 0.00095 ± 0.00016 for nucleotide diversity (π).

Table 4 shows the frequency of *POU1F1* gene genotypes (intron 5 and partial cds) in different rabbit breeds. BB, GB, and APRI breeds showed three genotypes compared to two genotypes in NZ breed. The frequency of *POU1F1* genotype 1 was high in BB (61.3%) and GB (70%) compared to APRI (31.2%) and NZW (33.7%) while the frequency of genotype 2 was high in APRI (53.8%) and NZW (66.3%) breeds compared to BB (22.5%) and GB (23.7%) breeds. The association of *POU1F1* genotypes with body weight revealed no significant differences between genotypes 1, 2, and 3 in BB, GB, and APRI breeds. The NZW breed displayed significant differences between genotypes 1 and 2. The mean value of body weight showed no significant differences between breeds.

**Table 4:** Frequency of the *POU1F1* gene genotypes (intron 5 and partial cds) in different rabbit breeds and their associated body weight

Breed	Genotyp1		Genotype2		Genotype3	
	Weight/kg Mean ± SD	Frequency %	Weight/kg Mean ± SD	Frequency %	Weight/kg Mean ± SD	Frequency %
BB	2.86 ± 0.33	61.3	2.88 ± 0.16	22.5	2.91 ± 0.50	16.2
GB	3.28 ± 0.31	70	3.3 ± 0.42	23.7	3.05 ± 0.18	6.3
APRI	2.93 ± 0.45	31.2	2.96 ± 0.28	53.8	2.99 ± 0.20	15
NZW	3.2 ± 0.34^A^	33.7	2.64 ± 0.37^B^	66.3	–	–

The rabbit breeds: Baladi Black (BB), Gabali (GB), APRI-line (APRI), and New Zealand (NZW)

The capital letters (A and B) in the same row significantly different at p ≤ 0.05

## Discussion

*Myf5* and *POU1F1* genes play important roles in the growth and development of mammals (AL-Khuzai and AL-Anbari 2018; Jaffar et al. 2019; Zhao et al. 2020; Zhang et al. 2022). Seong et al. (2011) suggested the *Myf5* and *POU1F1* genes as candidate genes for the growth traits; their results indicated a strong interaction between the two genes with growth and carcass traits in cattle.

### Genetic Characterization of the *Myf5* Gene and Its Association with Growth Traits

The *Myf5* gene plays a role in myogenic to induce an expression of muscle-specific genes during embryonic muscle development and can convert various non-muscle cells into muscle (Seong et al. 2011; Zhang et al. 2022).

All the amplified PCR products of *Myf5* gene exon 1 digested by *HindIII* resulted in two restriction fragments at 914-bp and 373-bp (Fig. 1B). The amplified PCR products of *Myf5* gene exon 2 digested by *HaeIII* showed two fragments at 564-bp and 371-bp (Fig. 3B). The results of PCR-SSCP technique and the sequence analysis of *Myf5* gene exon 1 of the four Egyptian rabbit breeds demonstrated two genotypes due to SNPs substitution (Figs. 1C and 2) while *Myf5* gene exon 2 of the four Egyptian rabbit breeds demonstrated four genotypes due to SNPs substitution (Figs. 3C). These results indicated that the PCR-restriction enzyme method by *HindIII* and *HaeIII* is not an appropriate technique to detect the genetic variation between the breeds under study. The genetic characterization of each breed was expressed through the appearance and frequency of genotypes. The *Myf5* gene diversity of the four breeds revealed that the APRI breed had one genotype of *Myf5* gene exon 1 and two genotypes of *Myf5* gene exon 2 while NZW breed showed two genotypes of *Myf5* gene exon 1 and three genotypes of *Myf5* gene exon 2. However, BB and GB breeds demonstrated two genotypes of *Myf5* gene exon 1 and four genotypes of *Myf5* gene exon 2 (Table 2 and 3).

The results of haplotype diversity and the nucleotide diversity (π) between breeds for the *Myf5* gene exon 1 and 2 indicated that the *Myf5* gene exon 2 was more diverse (0.03125 & 0.00366 ± 0.00079, respectively) compared to *Myf5* gene exon 1 (0.00475 & 0.00256 ± : 0.00034, respectively) (Fig. 4).

The genetic diversity of the *Myf5* gene exon 1 association with growth traits showed no significant differences in BB, GB, and APRI breeds while a significant difference was determined between genotypes of NZW breed (Table 2). The results indicated that *Myf5* gene exon 1 had no effect on the growth traits in BB, GB, and APRI breeds. The molecular marker for genetic improvement of the growth traits in the NZW breed could be genotype 1 in breeding selection.

The genotypes of *Myf5* gene exon 2 in their association with body weight detected significant differences in genotypes of BB and APRI breeds while no significant difference was detected in GB and ZNW breeds. These results suggested that the molecular marker for genetic improvement of growth traits in the BB breed could be genotypes (1 and 2) and genotype 2 for APRI in breeding selection (Table 2). Among/as for the breeds, the mean value of body weight for genotype 3 showed significant differences between BB and GB breeds while genotype 4 displayed a significant difference between BB and GB breeds and also between BB and NZW breeds (Table 2).

Genetic characterization of *Myf5* gene in previous studies on farm animals reported variation in the gene diversity and its association with quantitative traits between different species. In cattle, Seong et al. (2011); Sarti et al. (2014) and Nasr et al. (2016) reported a significant association between *MYf5* genotypes and body weight traits. The AB genotype was of a higher body weight than that for BB and AA genotypes. In pigs, the SNPs analysis of *MYf5* exons 1 and 2 revealed three genotypes with a significant effect on the meat quality (Liu et al. 2008). Another study on pigs by Nguyen and Nguyen (2013) suggested that the *MYf5*/*Hin1II* polymorphism was valuable for some carcass traits. In sheep, the genomic sequence analysis of *MYf5* in three Chinese Tibetan sheep breeds detected three polymorphisms with different frequencies among the breeds. The three mutations detected were significantly associated with the growth traits (Sun and Han 2016). In Grassland Short-Tailed sheep, two SNPs of *MYf5* promoter reported significant effect on the growth performance (Zhang et al. 2022). In rabbits, the polymerase chain reaction and DNA sequencing were used by Wang et al. (2017) to study the polymorphism in the *Myf5* gene and its association with meat quality. Their study reported molecular markers for meat quality improvement.

### Genetic Characterization of the* POU1F1* Gene and Its Association with the Growth Traits

The *POU1F1* gene is a transcription factor that regulates the expression of genes such as *GH*, *PRL,* and *TSH*. Previous studies on the *POU1F1* gene in different farm animals reported that there is a genetic association between diverse production traits and the diversity of the *POU1F1* gene among animal species (Dagong et al. 2016; Ma et al. 2017; Al-Khuzai et al. 2018; Jaffar et al. 2019; Işık et al. 2019; Zhu et al. 2019; Dorjay et al. 2021).

All the PCR products digested by *EcoRI* showed uncut fragments (Fig. 5B). The result indicated that the restriction site for *EcoRI* was not present in the amplified PCR products of *POUF1* (intron 5 and partial cds). The PCR-SSCP technique and sequence analysis of *POU1F1* gene of the four Egyptian rabbit breeds demonstrated three genotypes due to the SNPs substitution (Fig. 6). The results are in agreement with those of Wang et al. (2015). Their study on three breeds of Chinese rabbits reported the presence of three genotypes in the *POU1F1* gene (intron 5) with different frequencies among the breeds. The results suggested that the SNP substitution in the *POU1F1* gene (intron 5 and partial cds) may lead to different gene expressions in *GH*, *PRL*, *TSH*, and the gene itself which may affect the functions of these genes (Pan et al. 2008).

The *POU1F1* gene nucleotide diversity (π 0.00095 ± 0.00016) was low compared to *Myf5* gene exons 1 (0.00256 ± : 0.00034) and 2 (0.00366 ± 0.00079). The result suggested that the *POU1F1* gene is more conservative between breeds compared to *Myf5* gene (Luan et al. 2006).

The association of the *POU1F1* gene (intron 5 and partial cds) genetic diversity with growth traits showed no significant differences in BB, GB, and APRI breeds and among the breeds. The significant difference was only between genotypes 1 and 2 in the NZW breed. The results suggested that the *POU1F1* gene (intron 5 and partial cds) has no effect on the body weight traits in BB, GB, and APRI breeds, and the molecular marker for genetic improvement of the growth traits in the NZW breed is genotype 1 for breeding selection. The current results are consistent with those of a previous study on the association between *POU1F1* and growth traits in cattle (Pan et al. 2008), in goats (Ma et al 2017; Işık and Bilgen 2019; Zhu et al. 2019), and in sheep (Jaffar et al.^2019^) at birth weight. In rabbits, Wang et al. (2015) found that a novel SNP in intron 5 of the *POU1F1* gene was associated with meat quality traits.

## Conclusion

Genetic characterization and its association with quantitative traits in local breeds are important tools for the genetic improvement and sustainable management of animal genetic resources. The study suggested that the genetic diversity of *Myf5* and *POU1F1* genes is associated with the growth traits. The genetic diversity of *Myf5* gene exon 2 is a candidate marker for rabbit body weight traits. The *Myf5* exon 2 genotypes (1 and 2) of the BB breed, genotype 2 of *Myf5* exon 2 of the APRI breed, and genotype 1 of *Myf5* exon 1 and genotype 1 of *POU1F1* of the NZW breed can be considered candidate molecular markers associated with the improvement of the growth traits in these breeds.

## Data Availability

All data generated or analyzed during this study are included in this article.
